# Impact of Nitrogen-to-Phosphorus Substitution on End Group of the Y6 Nonfullerene Molecule for Organic Photovoltaics: A Comprehensive Modeling Study

**DOI:** 10.1021/acsomega.5c02254

**Published:** 2025-05-01

**Authors:** Leandro Benatto, Guilherme C. Q. da Silva, Matheus F. F. das Neves, João Paulo A. Souza, Luana Wouk, Lucimara Stolz Roman, Marlus Koehler, Graziâni Candiotto

**Affiliations:** † Instituto de Física, Universidade Federal do Rio de Janeiro, 21941−909 Rio de Janeiro, RJ, Brazil; ‡ Laboratoire ICB UMR 6303, Université de Bourgogne, 21078 Dijon, France; § Department of Physics, Federal University of Paraná, 81531−980 Curitiba, PR, Brazil; ∥ Institute of Physics, University of Brasília, 70919−970 Brasília, DF, Brazil

## Abstract

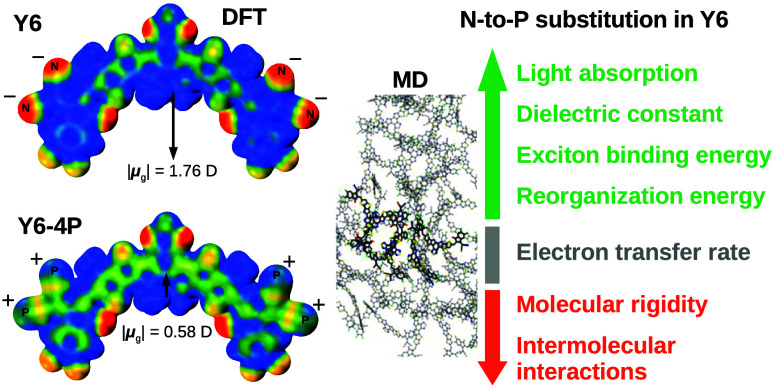

The N-to-P substitution in nonfullerene acceptors (NFAs) is an intriguing strategy to address environmental and economic concerns associated with the cyano (−C≡N) group. In this study, we performed a comprehensive computational investigation of N-to-P substitution in the end groups of the Y6 molecule, a benchmark NFA for organic photovoltaics (OPV). Using density functional theory (DFT), time-dependent DFT (TD-DFT), and molecular dynamics, we analyzed the effects on the electronic structure and intermolecular interactions. Our results reveal significant changes in intramolecular properties depending on the P atom’s position. The P≡C bond is longer than the N≡C bond, enhancing electronic delocalization, reducing both fundamental and optical gaps, and improving light absorption and exciton dissociation. However, P incorporation reduces the quadrupole moment, slightly weakening the intermolecular interactions and electronic coupling. Despite this, the electron transfer rate remains stable due to a compensation effect with intramolecular reorganization energy. Overall, our findings suggest that N-to-P substitution enhances the key optoelectronic properties of Y6, potentially benefiting OPV performance. This study provides valuable insights into the feasibility of this modification for organic electronics.

## 1 Introduction

Organic chemistry has long been characterized by the intricate manipulation of molecular structures to achieve desired properties.^1−3^ A promising strategy in the field of molecular design is the substitution of nitrogen (N) atoms with phosphorus (P).^4,5^ Nitrogen-containing functional groups have served as fundamental building blocks in organic synthesis and medicinal chemistry.^6,7^ However, the incorporation of phosphorus into molecular frameworks has raised interesting questions about the advantages of potential applications, since this substitution remains largely unexplored in the field of organic electronics.^8−10^ Because phosphorus has a radius larger than that of nitrogen, it will tend to increase intramolecular electronic delocalization, potentially leading to unique physical and chemical properties in P-based materials such as wide-bandgap structures. The versatility of phosphorus-based functional groups offers novel strategies for designing custom molecules with enhanced properties.^11^ As one enters the uncharted territory of nitrogen-to-phosphorus (N-to-P) substitution, computer simulations are a powerful tool for confirming the intricacies of these novel molecular architectures and predicting their behavior.^12−14^

In a worldwide context urging for energy transition, the application of phosphorus-substituted organic molecules in nonfullerene acceptors (NFAs) for organic photovoltaic (OPV) devices has a special appeal. NFAs have attracted immense attention for their exceptional electron-accepting capabilities, enabling the realization of highly efficient solar cells.^15,16^ In the search for more efficient NFAs, the modification of their end groups has generated interesting insights to improve their push–pull electronic structures.^17,18^ In this context, a highly toxic substance, malononitrile, used in the synthesis of the cyano (−C≡N) group (that is present in traditional NFAs) has raised environmental and economic concerns, motivating the search for a suitable substitute, such as, for example, −CH_3_, −CF_3_, −SO_3_H, and −NO_2_.^19−22^ Contrary to the cyano group, however, these substituents have a three-dimensional structure that increases the degree of molecular distortion. In addition, molecules containing the cyano group can release cyanide ions (CN^–^) under specific conditions. This release typically occurs through chemical reactions that break the carbon–nitrogen triple bond. Cyanide ions are highly toxic to living organisms because they interfere with cellular respiration by inhibiting key enzymes.^23^ Moreover, due to their high solubility in water, cyanide ions can easily contaminate rivers and water reservoirs, posing a significant threat to the environment.^24^ Due to the great difference between C≡N and C≡P triple bonds, the strategic incorporation of phosphorus atoms in NFA could offer distinct advantages compared to traditional NFAs with cyano in their structures and therefore deserve investigation. As mentioned in the review article by Chirila et al.,^25^ the C≡P triple bond of organophosphorus compounds is more related to the C≡C bond of alkynes than to the C≡N bond of nitriles, which is also reflected in their name, i.e., phosphaalkynes instead of phosphacyanides.

**Figure 1:**
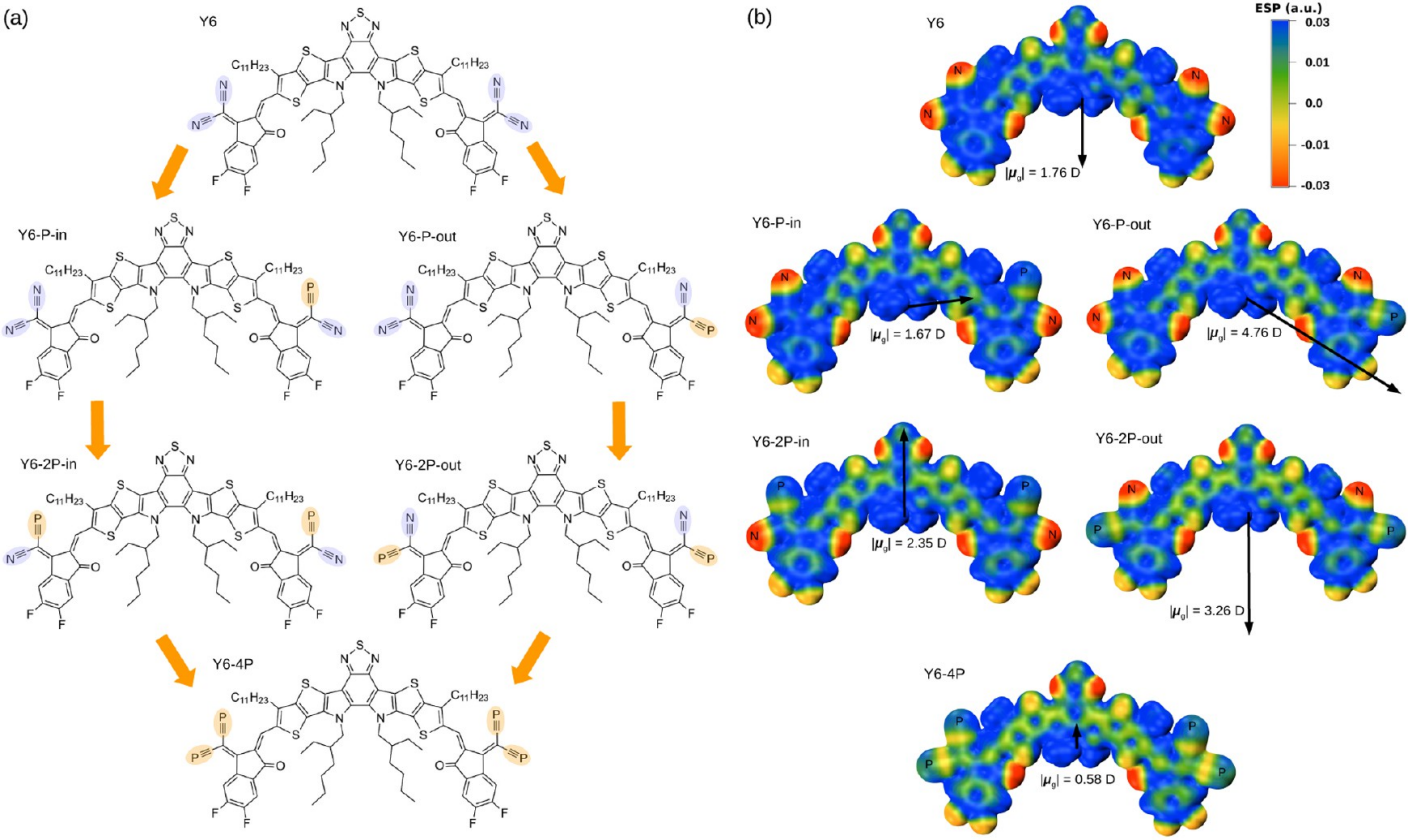
(a) Chemical structures of phosphorus-incorporated acceptor molecules. (b) Electrostatic potential (ESP) maps of the phosphorus-incorporated acceptor molecules. Inset: The black arrows represent the ground-state dipole moment vectors directed from negative to positive.

In this study, we employ state-of-the-art computational methods to explore the potential benefits of N-to-P substitution in the acceptor end groups of the Y6 molecule that present the A-DA′D-A-type structure (A and D are the electron acceptor and electron donor chemical units). The Y6 molecule, presented in Figure 1a, has become a standard NFA for organic photovoltaic devices due to its outstanding optoelectronic properties, including a low bandgap, strong near-infrared absorption, high electron mobility, and excellent morphological compatibility with donor materials.^26,27^ These characteristics contribute to efficient charge generation, transport, and collection, leading to high power conversion efficiencies in Y6-based acceptor devices.^15,28^ The substitution strategy aims to investigate the potential impacts of this modification on the optoelectronic properties of phosphorus-containing Y6 derivatives. To our knowledge, this study represents the first attempt to explore the integration of phosphorus atoms into acceptor materials featuring an A–DA′D–A configuration, a structural motif that has demonstrated unparalleled efficacy for photovoltaic applications.^29,30^ Quantum mechanical calculations, including density functional theory (DFT) and time-dependent DFT (TD–DFT), are utilized to predict changes in electronic structure, energy levels, and absorption spectra resulting from substitution. This approach is well-established in the literature and has been extensively applied in similar studies.^31−33^ Molecular dynamics (MD) simulations are also employed to probe the structural stability and intermolecular interactions of modified molecules within a realistic environment.^34,35^ The findings of this study provide valuable information about the feasibility of this innovative modification and its potential to enhance the optoelectronic properties of Y6-based materials.

In the following sections, we detail our methodology, present our computational findings, and discuss the broader implications of our discoveries for the field of organic electronics. Through our systematic exploration, our aim was to inspire researchers to consider the phosphorus substitution strategy as a potent tool for molecular design, fostering innovation and breakthroughs in photovoltaic applications.

## 2 Materials and Methods

### 2.1 Molecules Designation

The Y6 molecule has dual nitrogens within each end group. Our investigation encompassed five types of N-to-P substitutions, categorized into two asymmetric variants and three symmetric variants. Asymmetric molecules have only one P atom positioned inside (in) or outside (out) with respect to the molecular center. They are named here as Y6–P–in and Y6–P–out. Consequently, their symmetrical counterparts, which present two P atoms, one within each terminal group, were designated as Y6–2P–in and Y6–2P–out. Lastly, the exploratory scenario involving four N-to-P substitutions has been denoted as Y6–4P. A visual representation of the chemical structures is provided in Figure 1a.

### 2.2 DFT Methodology

The ground-state geometry optimization of the molecules and dimers and the Raman spectra of the molecules were calculated using the long-range corrected (LRC) hybrid functional ωB97XD^36^ along the 6–31G(d,p) basis set in vacuum.^37^ The range separation parameter (ω) of the functional ωB97XD was optimized by a gap-tuning procedure for the ground-state molecular geometry.^38^ Then, the electronic properties were calculated by using the same theory level. To reduce computational cost, we implement a commonly used simplification, replacing alkyl side chains with methyl groups.^39^ This simplification finds validation in the fact that alkyl side chains are typically incorporated to enhance the solubilization of the molecule without exerting a substantial influence on electronic properties. The vertical exciton binding energy of the molecules (*E*_b_ = *E*_fund_ – *E*_opt_) was calculated by subtracting the fundamental energy gap (*E*_fund_ = IP – EA) from the optical gap (*E*_opt_), where IP and EA are the vertical first ionization potential and electron affinity of the molecule, respectively.^40^ The intramolecular reorganization energy for electron transfer (λ_int_) was calculated using the adiabatic potential energy surfaces of the neutral and charged molecules considering the tuned ω.^41,42^ The outer component of the reorganization energy (λ_out_), which is much lower compared to the inner component,^43^ was set to 36 meV, a physically plausible parameter for the outer component.^44^ The singlet excited-state geometry optimization was calculated considering the tuned ω. The electronic coupling between LUMOs (β) was obtained with AOMix^45,46^ from a fragment orbital analysis.^47^ The electron transfer rate (*k*_e_) was estimated within the framework of the semiclassical Marcus/Hush theory^48^
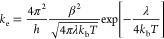
1where *k*_b_, *T*, and λ are the Boltzmann constant, temperature, and reorganization energy (λ = λ_int_ + λ_out_), respectively.

All DFT/TD-DFT calculations were performed using the Gaussian 16 package.^49^ Atomic partial charges were accurately determined using the restrained electrostatic potential (RESP) fitting method,^50^ which were computed using version 3.8 of the Multiwfn package.^51^

The relative dielectric constant (ε) of the acceptor molecules can be estimated using the Clausius–Mossotti equation
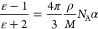
2where ρ, *M*, *N*_A_, and α are the density of the material, the molecular mass, the Avogadro constant, and the isotropic component of the molecular polarizability, respectively. A high dielectric constant is crucial to reduce the exciton binding energy, consequently minimizing the losses by charge carrier recombination.^14,52,53^ Note that in eq 2, ρ*N*_A_/*M* represents the inverse of the molecular volume (*V*_M_). Following the procedure established previously,^54^ both *V*_M_ and α will be obtained from MD simulations.

### 2.3 MD Methodology

Classical MD simulations were performed to mimic the evaporation procedure responsible for the formation of the NFA film. All simulations were carried out using the GROMACS^55−61^ code version 2023.2.^62^ The simulation protocol closely followed the same methodology described in a previous publication of a part of the group.^54^

Initially, 100 NFA molecules were inserted in a cubic box with a side diameter of 12.5 nm. Then, the box was filled with solvent molecules of chlorobenzene until a density of 1.1 g/cm^3^ was reached. This solvation procedure was carried out using the GROMACS built-in tools, and a previously equilibrated box of pure solvent was used as a pool of configurations. This initial box was then equilibrated at both the NVT and NPT runs. After these equilibration runs, cycles of equilibrium NVT simulations followed by the removal of the outermost solvent molecules were performed. For that, the simulation box was elongated in the *z*-direction with a final proportion of 1 × 1 × 7, so that the size of the vacuum region was 6 times the size of the initially equilibrated box.

The main goals of the MD simulations were (i) sampling molecular conformations for *ab initio* calculations of the polarizability; (ii) extracting the molecular volume; and (iii) investigating possible structural features of the NFA films formed after the evaporation.

The OPLS force field^63^ was used for the chlorobenzene and all NFA molecules, and the atom-type assignment was done with the help of the MOSDEF^64^ Python libraries (mBuild^65^ and Foyer^66^). It is important to note that the OPLS force field does not have parameters for the P ≡ C group. Consequently, the stretching and bending parameters for this group were independently accessed following a special procedure: first, reference length and angles were taken from the *ab initio* calculations, and force constants were approximated using the parameters for describing the nitrile group. Concerning the Lennard-Jones potential for the carbon atoms, the same parameters were chosen for the carbon in the N≡C moiety. The parameters of the P atom were taken from the universal force field (UFF).^67^

In order to obtain an ensemble of conformers to be applied in *ab initio* calculations, the last configuration after film formation was subjected to an additional energy minimization step. After that, each molecule was extracted from this configuration for subsequent calculations of the electronic structure.

The calculation of the molecular volume (*V*_mol_) also followed the same methodology described previously.^54^ It involves using the relation of fractional free volume (FFV) described in eqs 3 and 4, where FV, *V* and *N* are, respectively, the free volume, the total volume, and the number of the molecules of the simulation box. The GROMACS suite^68,69^ was also used to process the last frame of the trajectory in order to obtain FFV.
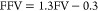
3
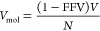
4

To investigate the possible influence of the chemical modifications to the molecular structure, conformational analyses were also carried out. The torsion between core and end groups were assessed by calculating the dihedral angle distribution in the same manner as Kupgan et al.^35^ Moreover, the coiling degree of the alkyl side groups was studied by analyzing the molecular radius of gyration (*R*_g_) and each chain end-to-end distance *d*_ee_. The intermolecular interaction energy was also assessed by comparing the energy of the film to the energy of an isolated NFA molecule.

Concerning the investigation of the differences between the structures of the films formed by each NFA molecule, three types of analyses were performed. The accessibility of the key atoms, that is, N and P involved in the modified triple bonds, was analyzed by calculating their solvent-accessible surface area. This was done using the default procedure of the GROMACS package.^69,70^ The obtained atomic accessibility values helped to reveal the formation of intermolecular pairs.

Moreover, clustering analysis and counting of the number of dimers formed in the film were performed. Two types of dimer counts were carried out: one considering all kinds of molecular pairs, and the other considering only the face-on pairs as defined by Kupgan et al.^35^ Both analyses were carried out using a Python script using the MDAnalysis library.^71,72^ These authors defined a face-on pair as a dimer of NFA molecules that present at least 25 pairs of atoms within a distance of less than 4 Å from each other. The pair counting considered only the backbone moiety. The DBscan^73^ method was used to detect the clusters. The minimum number of samples for a cluster was considered to be two, and the maximum distance between the samples was chosen to be 1 nm. The analysis was carried out considering the center of mass of the backbone atoms.

## 3 Results and Discussion

### 3.1 DFT Simulations

#### 3.1.1 Single Molecules

The electrostatic potential (ESP) distributions of the molecular models are mapped in Figure 1b. Nitrogen atoms in the end group of molecules have negative ESP values, while phosphorus atoms have positive values. Because N and P form a triple bond with C, this phenomenon can be explained by looking at the electronegativity of these elements. In particular, the carbon atom has an electronegativity of 2.55, an intermediary between nitrogen, 3.04, and phosphorus, 2.19, which justifies the phenomenon. Due to the electron deficiency of the C sp atom, it can act as an electrophile, promoted by the high electronegativity of nitrogen and the high dipole moment in the triple bond in the nitrile group. Considering the partial charges in the unit of electrons associated with these atoms (*q*_N_, *q*_C_, and *q*_P_), an interesting general effect can be observed. For the N≡C interaction, the partial charges are almost equal, with *q*_N_ ≈ −0.45 and *q*_C_ ≈ 0.44. In contrast, for the P≡C interaction, there is a clear effect where *q*_C_ becomes negative, *q*_C_ ≈ −0.1, while *q*_P_ is close to zero and positive, *q*_P_ ≈ 0.02. Since *q*_N_, *q*_C_, and *q*_P_ vary slightly between molecules, their values have been given as approximations to facilitate understanding. Regarding the length of the triple bond P≡C, we have a calculated value of 1.55 Å, which is in good agreement with previous theoretical and experimental results.^12,74^ Notably, this value is longer than the triple bond length observed for N≡C, which is 1.16 Å, as found in this study and in other investigations.^75^ This behavior is expected since the atomic radius of P is larger than the atomic radius of N (a higher number of inner electronic shells).

The change in the ground-state charge distribution due to molecular modification influences the dipole moment vector (μ_g_-directed from negative to positive); see Figure 1. Specifically, the symmetrical configuration of the Y6 molecule relative to a reflection plane σ_v_ is characterized by a centrally oriented dipole moment with a magnitude of |μ_g_| = 1.72 D. Conversely, molecular structures that break this symmetry induce changes in both the amplitude and orientation of the dipole moment. Notably, this symmetry break results in the dipole moment aligning toward the more positively charged end group. This trend is particularly pronounced in the case of the Y6–P–out molecule, wherein the positive charge accumulates at the molecular tip, resulting in a substantial dipole moment magnitude of |μ_g_|= 4.76 D. Among symmetrically modified molecules, the centralized orientation of μ_g_ is preserved. Note that to the Y6–2P–out molecule, the orientation of μ_g_ remains the same, but its magnitude increases to 3.16 D in relation to Y6. In contrast, the molecules Y6–2P–in and Y6–4P exhibit an inversion in the orientation of μ_g_, accompanied by an increase in the dipole’s magnitude for the former and a decrease for the latter. Appealing to the Debye equation, it is expected that an increase in the molecule’s dipole moment would typically result in a corresponding enhancement of its dielectric constant.^3,76^ The results in Section 3.2 partially confirm this general trend, as the MD calculations indicate that, indeed, the dielectric constants of Y6–P–out and Y6–2P–out are higher than those of Y6.

**Figure 2:**
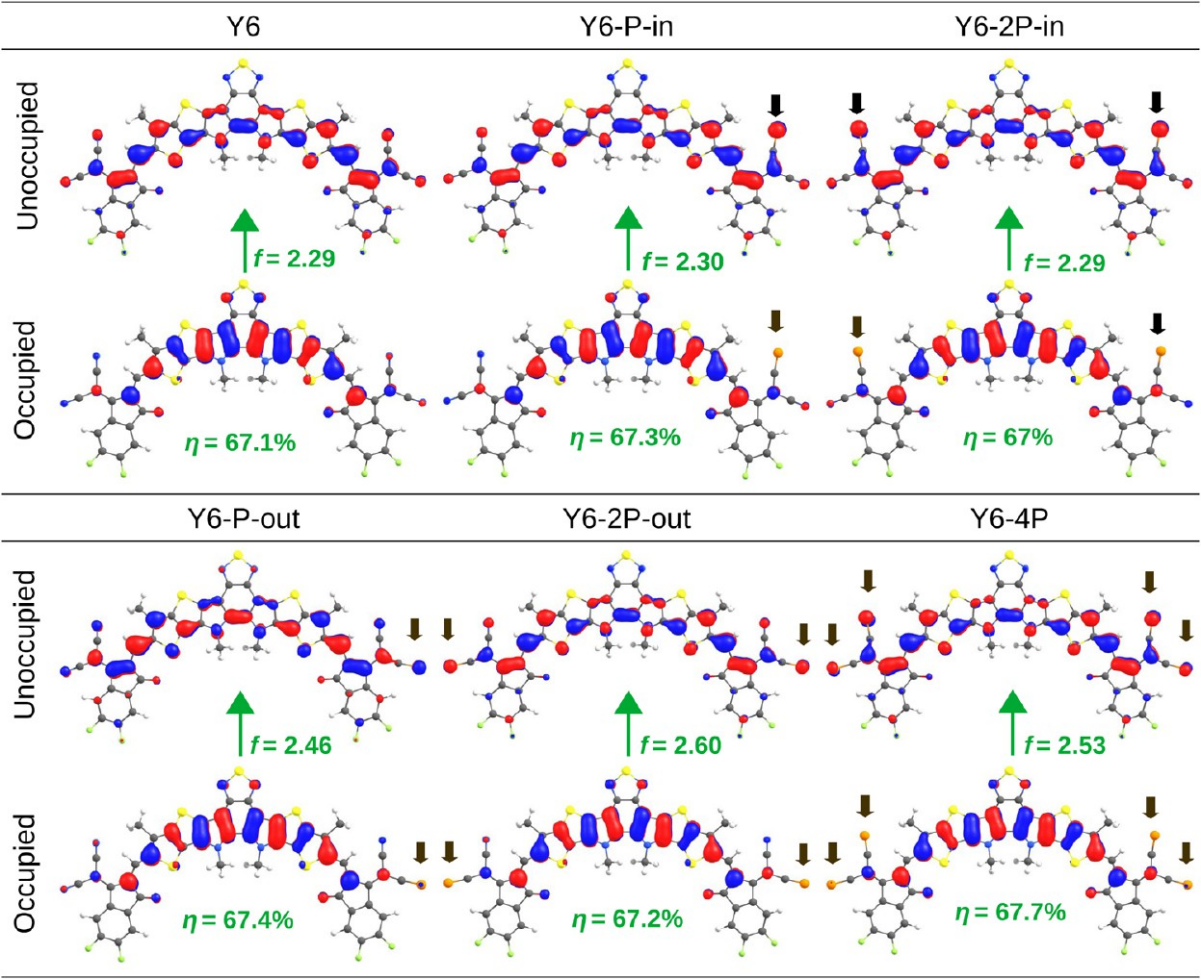
Spatial distribution of natural transition orbitals (isovalues 0.03 au) of the phosphorus-incorporated acceptor molecules. Inset: The green arrows indicate the S_1_ transition, and the black arrows indicate the position of the phosphorus atoms. The oscillator strength (*f*) of the S_1_ excitation and the overlap between the two orbitals (η) are provided.

**Figure 3:**
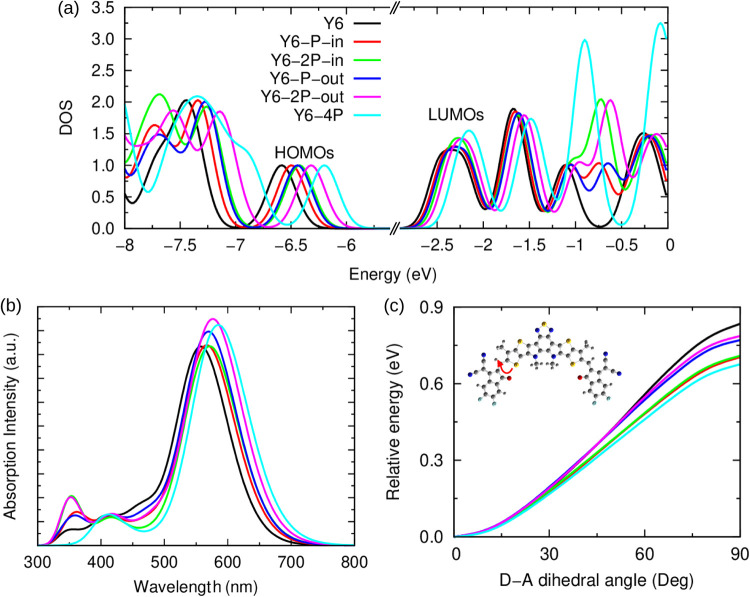
Properties of the phosphorus-incorporated acceptor molecules simulated in vacuum. (a) Density of states (DOS). (b) Absorption spectrum. (c) Relative molecular potential energy vs the dihedral angle between the central fused-ring core and acceptor end group. The red arrow indicates the dihedral angle between the central fused-ring core (D) and the acceptor end group (A) that changes in the potential energy scan.

Figure 2 shows the spatial distribution of the natural transition orbitals of the first singlet state (S_1_). The black arrows indicate the positions of the phosphorus atoms. At the sites where phosphorus is inserted, there is a visible change in the unoccupied molecular orbital. The electronic density in the region increases, thereby enhancing electronic delocalization upon photon absorption. The longer P≡C bond length compared to that of N≡C also helps to increase the electronic delocalization. This effect produces a red shift in the absorption spectrum, as can be seen in Figure 3b. The oscillator strength (*f*) of the S_1_ excitation increases with the number of substitutions of phosphorus atoms, which tends to improve the efficiency of visible light harvesting. This characteristic is also apparent in the absorption intensity of Figure 3b, where the Y6–2P–out molecule (which has the stronger *f* = 2.6) shows the highest absorption peak around 580 nm compared to the other molecules. Regarding the overlap between the natural transition orbitals (η) in Figure 2, a certain stability is observed with values around 67%. Hence, the red shift in Figure 3b cannot be attributed to an enhancement of the push–pull effect but rather to orbital delocalization induced by P substitution.

Figure 3a presents the molecule’s density of states (DOS). The phosphorus-incorporated molecules have a narrow HOMO–LUMO gap (or fundamental gap, *E*_fund_ = IP – EA) compared to that of Y6 due to a significant stabilization of the HOMO energy and a slight destabilization of the LUMO energy. Considering the order of the molecules in the legend of Figure 3a, *E*_fund_ is 4.19, 4.14, 4.13, 4.11, 4.06, and 4.03 eV, respectively. The dihedral angle between the central fused-ring core (DA′D) and acceptor end group (A) is close to zero in ground-state geometry for all molecules. This suggests that the molecular modification preserves the flat geometric conformation, which tends to favor an efficient π–π intermolecular stacking. Improved intermolecular interactions are advantageous for the charge transfer and transport in thin films.

In this context, a high torsional energy barrier between the DA′D and A parts of the NFAs is important to hinder molecular fragmentation and improve molecular photostability.^77^Figure 3c evaluates the relative potential energy as a function of the dihedral angle between the DA′D core and the A end group of the substituted NFAs. It is clear that the rotational energy barrier decreases with phosphorus incorporation. This reduction is marginal when the P insertion occurs in the outward positions, as observed in molecules Y6–P–out and Y6–2P–out. The small decrease of the rotational energy barrier upon phosphorus incorporation can be attributed to the lower stiffness of the C–C and C=C bonds that connect DA′D and A after the introduction of P. Bond length alternation (BLA) in the D–A region slightly increases with the substitution (except for Y6–P–out), as shown in Table S1. The small softening of the DA′D–A barrier then follows a decrease in the degree of hybridization of these bonds.

**Figure 4:**
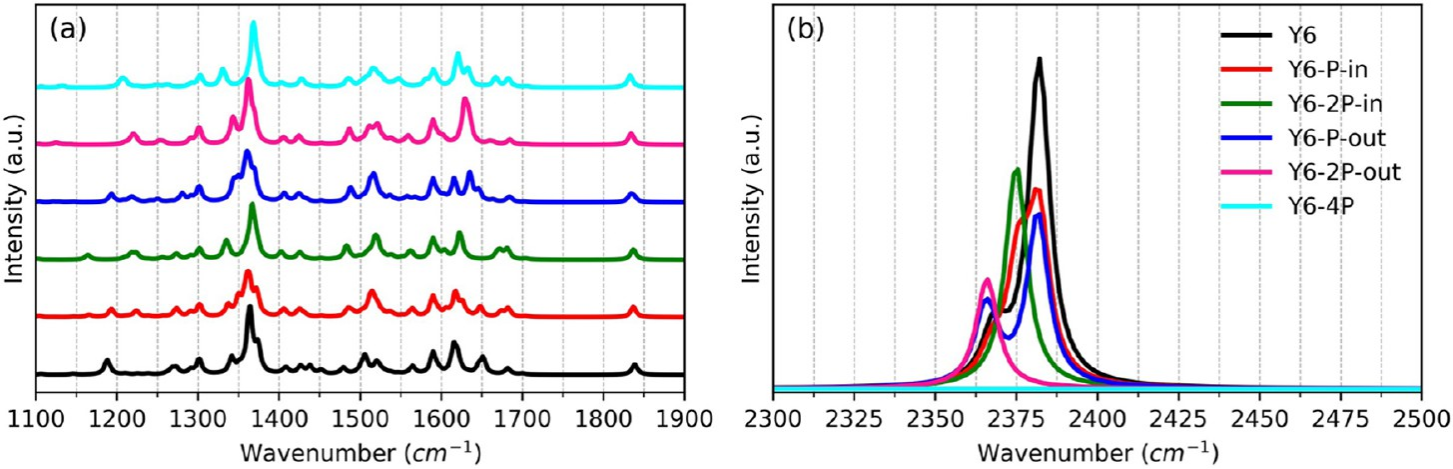
Raman spectra for all samples (a) from 1100 to 1900 cm^–1^ and (b) from 2300 to 2500 cm^–1^.

To gain a deeper understanding of the impact of N-to-P substitution, the molecule’s Raman spectra were also simulated. Here, we focus on the most significant findings regarding P substitution. For the interested reader, a more detailed analysis of the spectra is developed in the SI file. The Raman spectra in the range from 1100 to 1900 cm^–1^ and from 2300 to 2500 cm^–1^ are shown in Figure 4(a,b), respectively. The main features of these spectra are in accordance with those experimentally reported in recent literature.^78,79^ One can notice that characteristic bands for C_α_–C_β_ asymmetric stretching and C_α_–C_β_ symmetric stretching are found in the interval from 1510 to 1570 and 1450 cm^–1^, respectively. After P inclusion, the band associated with symmetric mode has a very low intensity that might be due to mass effect, electronic conjugation, and/or different bond stiffness that can interfere with specific vibrational modes. The higher mass of the P atoms might be unfavorable to promote polarizability variations upon symmetric vibrations decreasing the Raman response of those modes. For asymmetric modes, the intensities are similar but are blue-shifted, indicating that this specific bond is stiffer than without phosphorus inclusion, since P is heavier than N and does not represent the carbon bonds that connect DA′D and A parts of the Y6 molecule.^80,81^ The SI file provides an additional graph displayed in Figure S1 from 300 to 1100 cm^–1^ and a description of other bands of interest.

New vibrational and stretching bands (relative to Y6) appear in phosphorus-substituted molecules at 550, 1550, and 1580 cm^–1^ regions. These bands originate from carbon bonds that are especially susceptible to molecular geometry. In this region, a band at 1560 cm^–1^ vanishes in the Y6–4P molecule. The same can be observed with a Raman shift around 1340 cm^–1^. Relative to the 1360 cm^–1^ band of Y6, there is a blue (red) shift for Y6–P–in (Y6–P–out). The Y6–4P also shows a similar blue shift in this region, which can be associated with the elongation of BLA between the bonds C–C and C=C that connect the DA′D and A regions of the molecules after P inclusion. This is in accordance with the results presented in Table S1. Bands around 1400–1500 cm^–1^ are red-shifted after P substitution, and it can be associated with carbon bonds outside DA′D–A connection bonds. The same shift in the band around 1840 cm^–1^ of the Y6 molecule is observed after P substitution, which indicates a reduction in the vibrational frequency, likely due to changes in bond stiffness following mass effects. Peaks at higher wavenumbers in the region between 2300 and 2500 cm^–1^ can be linked to C≡N vibrations. This band is totally suppressed in the Y6–4P sample, which can be connected to the C≡P substitution of the original triple bond.^82,83^ A clear distinction between bands related to C≡P vibrations and bands already present in the substituted Y6 molecule is difficult in the 1200 cm^–1^ region. For instance, the peak around 1220 cm^–1^ can be associated with vibrations of the C≡P bonds^80^ and/or vibrations of the ketone group present in the Y6 molecule.^31^ Yet, it is notorious in Figure 4 that this band is more intense for samples with phosphorus atoms.

Since the simulated molecules are expected to be applied in photovoltaic devices, it is important to study molecular parameters related to exciton diffusion. Kashani et al.^84^ demonstrated that the high rigidity after electronic excitation is one of the reasons behind the long exciton diffusion length in Y6. One of the methods employed by these authors to estimate the molecular stiffness was to calculate the energy difference between the vertical absorption (*E*_S_0_→S_1__) and the vertical emission (*E*_S_1_→S_0__) energies. This difference estimates the intramolecular contribution to the Stokes shift, Δ*E*_rel_^int^ = *E*_S_0_→S_1__ – *E*_S_1_→S_0__. The difference tends to be lower for more rigid molecular structures. In Table S2 and Figure S2, we present the Δ*E*_rel_^int^ results for the molecules studied here. It is observed that the molecules Y6–P–in, Y6–P–out, and Y6–2P–out exhibit an Δ*E*_rel_^int^ value of 0.24 eV, identical to that of Y6, while the molecules Y6–2P–in and Y6–4P show slightly higher values of 0.25 and 0.27 eV, respectively. Therefore, in view of the intramolecular contribution, the molecules with phosphorus present molecular rigidity after electronic excitation similar to that estimated for Y6. Taking into account the intramolecular reorganization energy, molecules containing phosphorus have reduced values compared to Y6 (see Table 1 and Figure S3), which is important to lower the energy barrier for electron transfer, benefiting charge transport.^85^

**Table 1:** Molecular Quadrupole Moment (*Q*_π_ in Debey Å), Intramolecular Reorganization Energy (*λ*_int_ in eV), Electronic Coupling of LUMO Orbitals (β in meV), and Electron Transfer Rate (*k*_e_ in 10^12^ s^–1^)

	M		J		H		C	
molecules	*Q*_π_	λ_int_	β	*k*_e_	β	*k*_e_	β	*k*_e_
Y6	75.39	0.28	57.1	0.451	103.4	1.48	123.0	2.01
Y6–P–in	70.38	0.25	48.2	0.452	85.7	1.43	120.6	2.83
Y6–2P–in	63.95	0.24	48.2	0.507	71.2	1.11	120.7	3.18
Y6–P–out	53.34	0.26	44.8	0.348	92.0	1.47	113.4	2.23
Y6–2P–out	30.02	0.25	33.6	0.220	79.8	1.24	106.3	2.20
Y6–4P	21.87	0.23	29.8	0.218	56.9	0.793	103.8	2.64

**Figure 5:**
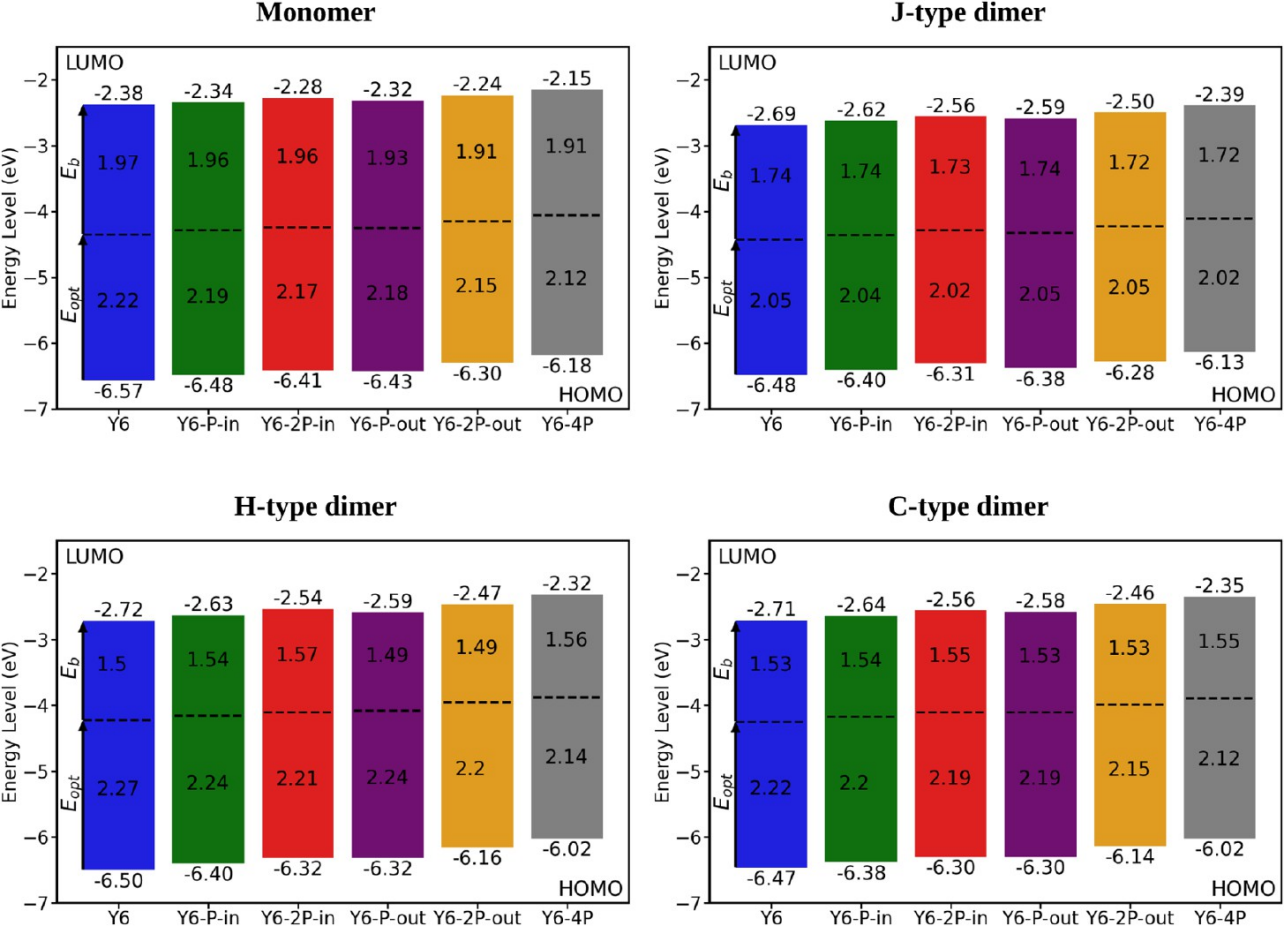
Energy level diagram for monomers (M) and dimers (J, H, and C). The dotted lines indicate the optical gap energy.

Figure 5 (top left) shows the energy levels along with the respective singlet optical gap and exciton binding energy for monomers. As previously discussed, the incorporation of phosphorus atoms into molecules leads to a reduction of the fundamental gap. The same trend is observed for the optical gap, albeit to a lesser extent. When these two effects are considered together, they collectively contribute to weakening the exciton binding energy, as expressed by the equation *E*_b_ = *E*_fund_ – *E*_opt_. The reduction of the exciton binding energy allows the use of photovoltaic blends with lower driving force, which minimizes voltage losses.^86^

It is well-established that the strength of the exciton binding energy can also be reduced by intermolecular interactions that play a pivotal role in enhancing electronic delocalization.^14,87,88^ Moreover, it is known that quadrupole–quadrupole interactions significantly contribute to improved intermolecular packing.^89^ With these effects in mind, we evaluated the molecules’ quadrupole moment. The results in Table 1 indicate that P incorporation leads to a reduction of *Q*_π_, the quadrupole component along the π–π stacking direction. The observed decrease of *Q*_π_ might be related to a more symmetrical charge distribution along the π–π stacking direction.^90^

#### 3.1.2 Molecular Dimers

To further explore intermolecular interactions, we conducted simulations of molecular dimers with a focus on three types, namely, J, H, and C aggregates. Those structures are predominant in Y6 thin films.^91,92^ J–type dimers have a head-to-tail orientation of the individual transition dipoles (↔↔), while H and C have a sandwich-type arrangement of these dipoles .^93,94^

**Figure 6:**
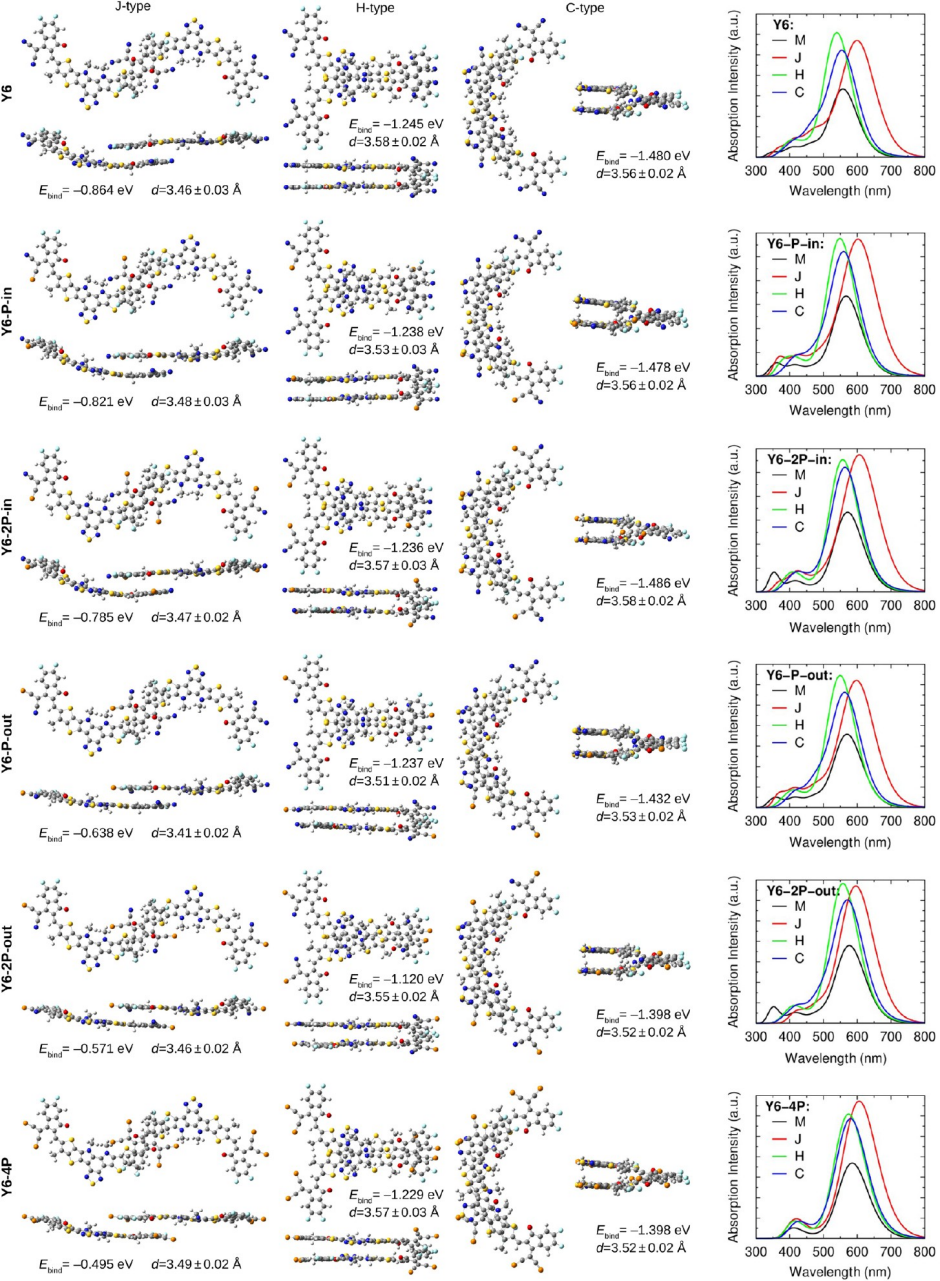
Optimized structure of dimers and calculated values of binding energy (*E*_bind_), packing distance (*d*), and absorption spectra. In the figure, we present the dimers in front and side view.

Checking first the effect of intermolecular interactions on the absorption spectra (Figure 6), the characteristic red shift (corresponding to a smaller optical gap, Figure 5) is observed for the J-type dimers.^95^ Regarding the effect of intermolecular interactions on exciton binding energy, *E*_b_ (Figure 5), both H- and C-type dimers exhibit a more pronounced decrease in this energy in comparison to the monomer. This is a consequence of the absorption shift to higher optical energies (*E*_opt_), which is characteristic of these kinds of dimer configurations. Furthermore, in a comparison of isolated molecules with dimers, the decrease of *E*_b_ is smaller for the modified molecules than for Y6, as shown in Figure 5. This is because the introduction of phosphorus atoms lowers intermolecular interactions and, consequently, the intermolecular electronic delocalization.

The simulation of the dimers shows that the intermolecular interaction (named binding energy, *E*_bind_) wanes with the insertion of phosphorus atoms, as seen in Figure 6. The electronic coupling of LUMO orbitals (β), which is responsible for electron transport, also reduces (see Table 1). As previously mentioned, these effects may be related to a decrease of *Q*_π_. Applying the Marcus formalism (eq 1), we were able to estimate the impact of molecular modifications on the electron transfer rate (*k*_e_). Interestingly, *k*_e_ undergoes small changes with molecular modifications. This relative stability of *k*_e_ arises from a partial compensation effect because both β and λ_int_ decrease with the P substitution. For example, looking at the J-type dimer, Y6–2P–in exhibited the highest *k*_e_, while the Y6–4P dimer displayed the lowest. However, H and C aggregates have the fastest *k*_e_ rates produced by higher values of β from greater orbital’s overlap.

Overall, DFT results for dimers indicate that the improvements in intramolecular properties observed with the introduction of phosphorus atoms are attenuated by poorer intermolecular properties. In order to check whether these features remain true on larger scales, it is necessary to go beyond limited DFT simulations. Consequently, MD calculations were employed to calculate the molecular packing and orientations of larger systems.

### 3.2 MD Simulations

**Figure 7:**
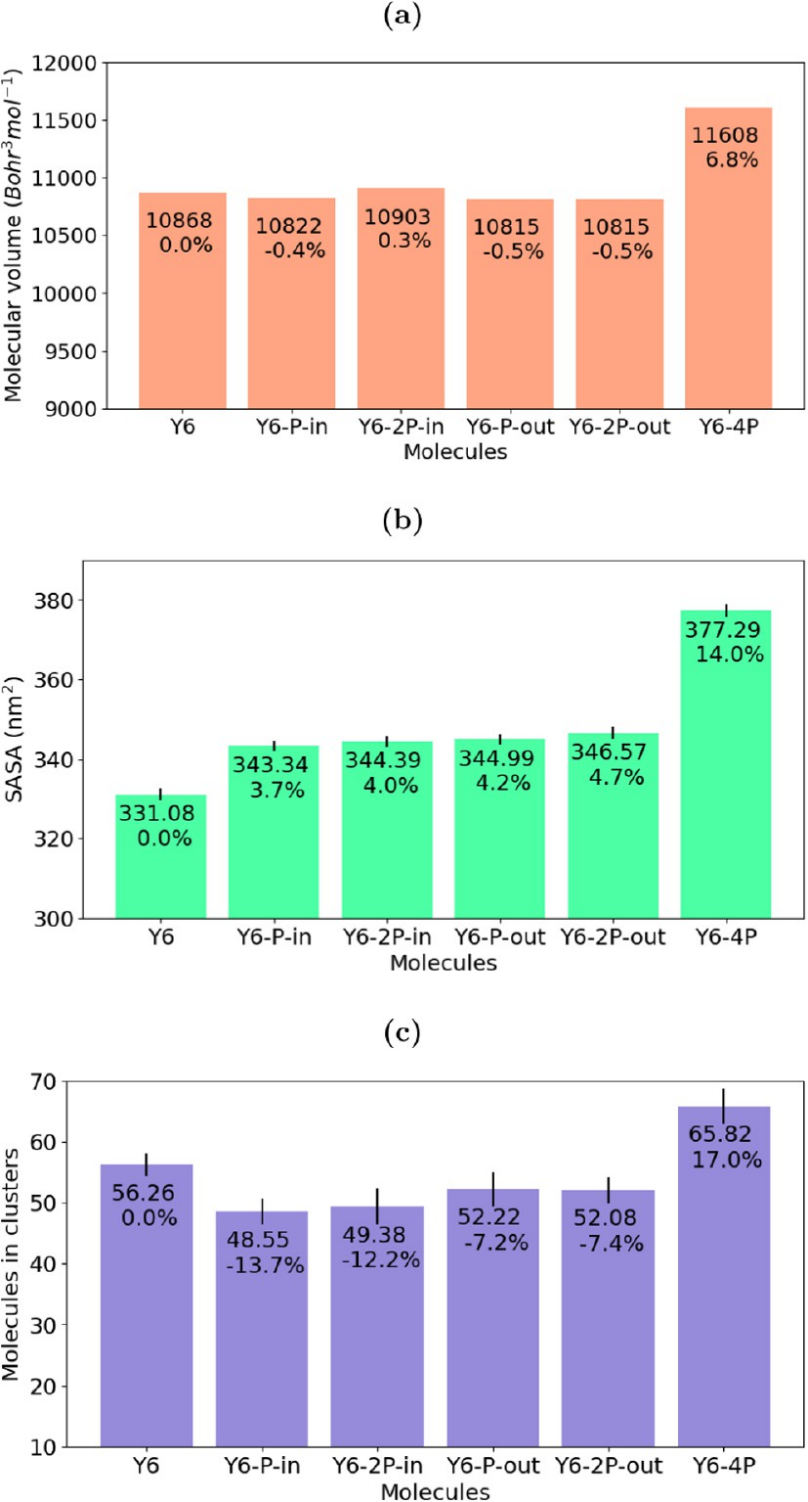
(a) Molecular volume (*V*_M_); (b) average solvent-accessible surface area (SASA) of the key N and P atoms; and (c) average number of molecules in clusters. Inset: respective values and percentage of variation in relation to Y6.

MD calculations were first employed to find the molecular volume (*V*_M_) of the materials in order to derive the dielectric constant (ε) from the Clausius–Mossotti eq 2. Later, MD results were also applied to study the possible films’ morphology. As illustrated in Figure 7a, the molecular volume (*V*_M_) remained relatively stable across the P modifications studied; the exception is the Y6–4P molecule. It exhibited a *V*_M_ increase of nearly 7%, which is expected due to the larger size of the P atom compared to N. For instance, the Bondi’s van der Waals radii (*r*_vdW_)^69^ of the P atom is about 16% larger than the radii of the N atom. However, the fact that only the Y6–4P showed a significantly higher *V*_M_ may indicate that other structural or morphological factors can partially compensate for the bigger volume of the P atoms for lower concentration of phosphorus substitutions. One of those factors might be a denser molecular packing during the film formation or changes in the conformer population. In the sequence, we further explore the MD results in order to explain the *V*_M_ behavior.

**Figure 8:**
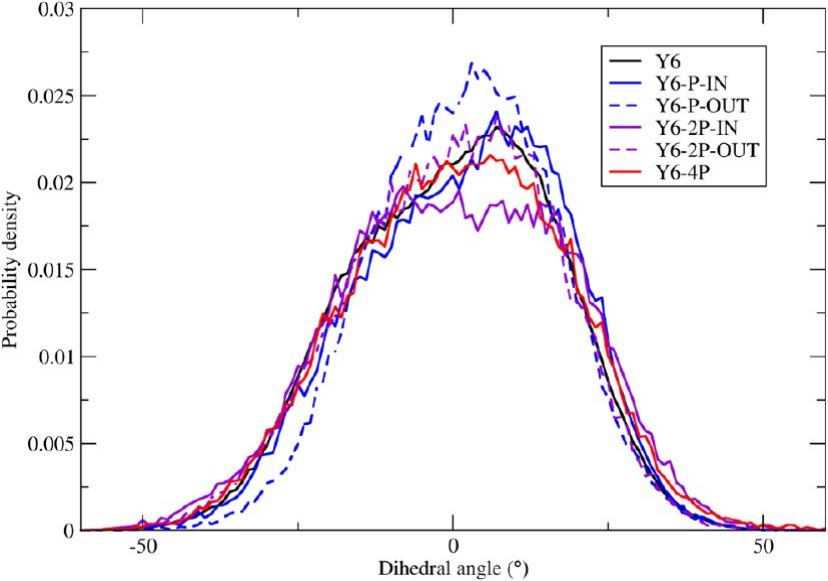
Dihedral angle distribution of the torsion between central fused-ring core and acceptor end group.

In principle, N-to-P substitution can affect the rigidity of torsions involving central and terminal groups of the molecule (as seen in Figure 3c). Figure 8 displays the angle distributions of the aforementioned torsions for each system. The results are in line with a similar analysis available in the literature.^35^ No significant changes were observed in the dihedral angle distribution when N atoms were replaced by P. These findings indicate that the small differences observed in the DFT calculations with isolated molecules (Figure 3c) are mitigated by intermolecular interactions.

The chemical change of the molecule could also exert an influence on the coiling of the side groups. This would affect the compactness of the molecule and, thus, its volume. Table 2 presents both the average radius of gyration (*R*_g_) and the end-to-end distance of the alkyl chains (*d*_ee_). As can be seen, no significant changes of these parameters are observed for the different systems.

**Table 2:** Radius of Gyration (*R*_g_), End-to-End Distance (*d*_ee_), Number of Pairs, Number of Face-on Pairs, Number of Atomic Contacts, Intermolecular Interaction Energy (*E*_inter_), Intermolecular van der Waals Energy (*E*_inter–vdW_), and Intermolecular Electrostatic Energy (*E*_inter–Coul_)

system	*R*_g_ (Å)	*d*_ee_ (Å)	N. pairs	N. face-on pairs	N. contacts	*E*_inter_ (kJ/mol)	*E*_inter–vdW_ (kJ/mol)	*E*_inter–Coul_ (kJ/mol)
Y6	8.94 ± 0.01	11 ± 2	45 ± 2	28 ± 2	60 ± 20	–269 ± 2	–256.2 ± 0.7	–5.1 ± 0.6
Y6–P–IN	8.94 ± 0.01	11 ± 2	44 ± 2	27 ± 2	60 ± 30	–270 ± 1	–258 ± 2	–6 ± 1.0
Y6–2P–IN	8.89 ± 0.01	11 ± 2	48 ± 2	23 ± 2	60 ± 30	–259 ± 2	–249 ± 1	–4.8 ± $0.6
Y6–P–OUT	9.03 ± 0.01	11 ± 2	44 ± 2	26 ± 2	60 ± 20	–266 ± 2	–250 ± 1	–4.6 ± 0.5
Y6–2P–OUT	9.00 ± 0.01	11 ± 2	51 ± 2	25 ± 2	60 ± 30	–267 ± 2	–258. ± 2	–2.9 ± 0.6
Y6–4P	9.02 ± 0.01	11 ± 2	53 ± 2	28 ± 2	50 ± 20	–259.4 ± 0.8	–254 ± 1	–1.5 ± 0.5

The intermolecular energy values are also displayed in Table 2. It is possible to see that van der Waals interactions dominate over the Coulombic contributions for all systems. In addition, only very subtle changes in the energies are observed. This result indicates that the better packing of Y6 and Y6–4P over the other systems (as it will be discussed below) might be caused by their higher degree of molecular symmetry that tends to ease ring stacking.

A detailed investigation of the morphological structure of the films derived from MD calculations is then necessary to clarify this issue. One quantity that is roughly correlated to the system packing is the solvent accessibility of the atoms. Figure 7b displays the calculated average solvent-accessible surface area (SASA) of the key P and N atoms in each molecule. When comparing the solvent accessibility by means of the SASA calculation, it is possible to observe that only Y6 and Y6–4P differ within the set. This result can be explained by taking into account the film structure and the atomic sizes of the atoms. Systems with a more tight molecular packing present smaller free volume and SASA. The molecular volume calculated by means of eqs 3 and 4 depends on the contributions of terms involving the atomic size and free volume.^96^ Based on this reasoning, it is possible to explain both the accessibility and molecular volume trends by a combined effect of changes in the atomic size and film structure, as it will be discussed below.

As mentioned previously, the P atom presents a larger *r*_vdW_ than N. Since the SASA analysis is also based on the same *r*_vdW_ set as the one used for free volume calculations, it is expected that substitution with P atoms would increase solvent accessibility. However, an appreciable increase in the SASA value was observed only in the fully substituted case. All of the mono- or disubstituted compounds showed only a mild increase in accessibility. Thus, the SASA analysis can distinguish systems with or without the P atoms, but it is not able to differentiate between systems with a single modification or two modifications.

**Figure 9:**
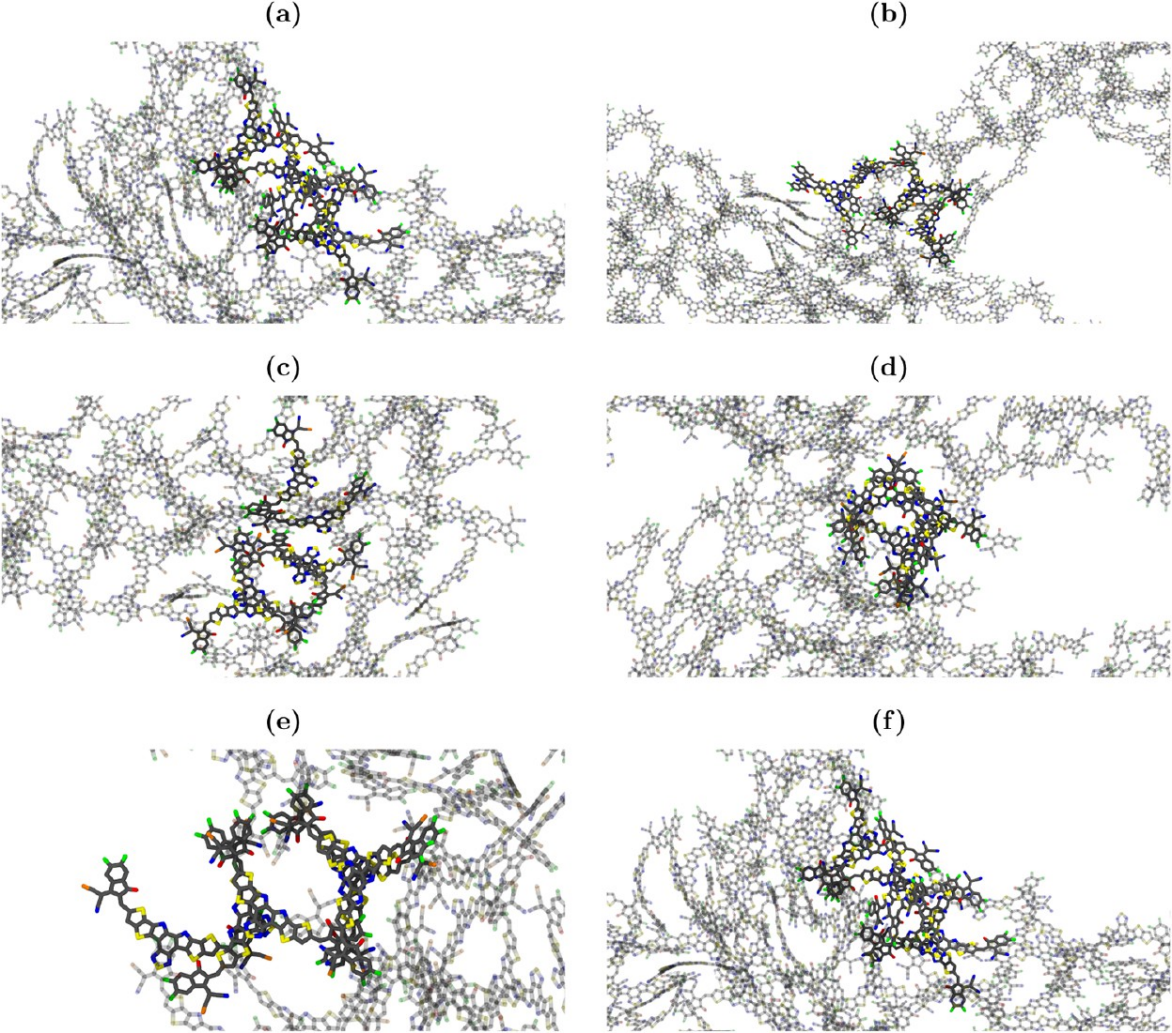
MD simulation snapshot of largest clusters: (a) Y6; (b) Y6–P–in; (c) Y6–2P–in; (d) Y6–P–out; (e) Y6–2P–out; (f) Y6–4P. Only the backbones of molecules are displayed.

A careful analysis of the molecule’s packing behavior suggests an explanation for this trend. The number of atoms in close contact, as well as the number of unclassified and face-on pairs, are displayed in Table 2. As discussed by Kupgan et al.,^35^ a number of 25 contacts reflects two 5-membered rings on a face-on pair configuration. As can be seen for the values of the face-on pairs and atomic contacts, all systems present a similar packing degree with an average stacking of two 5-membered for each pair (±15-atom ring). Snapshots of the simulations (Figure 9) revealed that the majority of this stacking happens between the rings within the region connecting one end group and the core group (this kind of π–π stacking was indeed observed experimentally for both single crystals and spin-coated films of Y6^91,97,98^).

In other words, the most likely orientation between a pair of molecules tends to reduce the accessibility of only one pair of key atoms located at the molecule’s end group. This packing pattern is not affected by the position of the P substitution. As a consequence, the potential increase of the accessibility associated with the larger size of P is reduced with partial phosphorus substitutions. The exception is the Y6–4P molecule, where there are always two P atoms at each molecular end group to enhance the SASA values.

Another important structural aspect that influences the accessibility of key atoms (consequently, the molecular volume) is the occurrence of molecular clusters. Hence, the number of molecules that take part in clusters, as shown in Figure 7, is another criterion to quantify the packing efficiency of the systems. The inclusion of the P atom seems to reduce the number of clustered molecules, regardless of the position of P atom addition. On the other hand, again the behavior is different for complete substitution with P≡C so that the number of molecules belonging to a cluster is considerably higher for the Y6–4P molecule.

To summarize, compared to the systems with one or two substitutions, Y6 presents atoms with smaller *r*_vdW_, but this size effect is compensated by a tighter packing. Considering the substituted molecules, the contribution due to the size of the P atom auditioned to the end group is lessened by the formation of intermolecular pairs. As a result, systems with only one or two substitutions present similar molecular volumes as Y6. The Y6–4P, however, has a tight molecular structure together with more exposed P atoms with greater *r*_vdW_. Hence, it presents a higher molecular volume. The same reasoning is valid to explain the SASA values. The more packed molecules tend to decrease the exposure of the bigger atoms attached to the edges. In the opposite direction, larger *r*_vdW_ tends to contribute more to increasing SASA values. The similar structure of systems with only one or two modifications results in indistinguishable SASA values due to intermolecular pair formation. Naturally, Y6 has the lowest SASA associated to an effective packing and smaller atoms, while the Y6–4P has the largest SASA from the enhanced contribution of large exposed P atoms at the molecule’s end groups.

**Figure 10:**
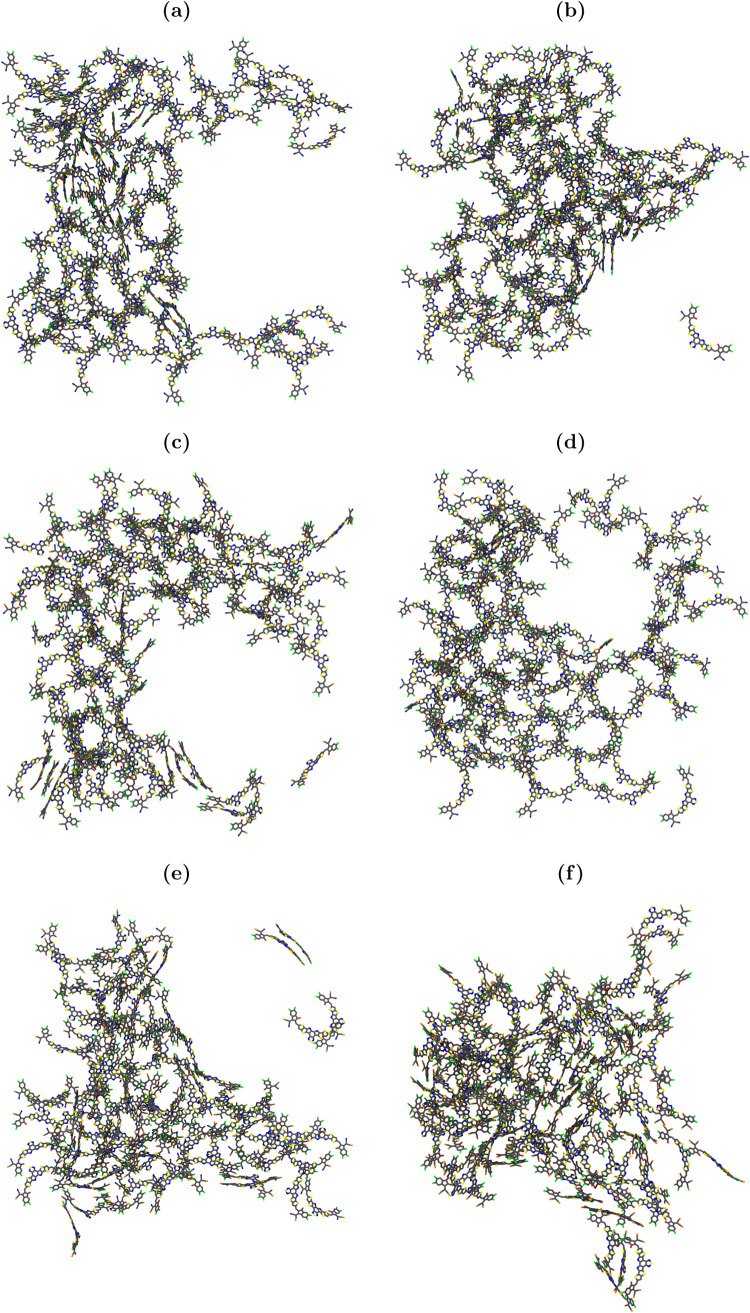
Snapshot of simulation box: (a) Y6–P–IN; (b) Y6–2P-IN; (c) Y6–P–OUT; (d) Y6–2P-OUT; (e) Y6–4P; and (f) Y6. Only the backbones of molecules are displayed.

It is also interesting to show the morphological structure of the resulting film after solvent evaporation on a larger scale. Figure 10 displays snapshots of the MD simulation box. It is possible to observe a defective elliptical framework resulting from the aggregation of intermolecular pairs. The formation of such aggregates is driven by the π–π stacking between the rings within the region connecting the core and terminal groups.^91,97,98^

**Figure 11:**
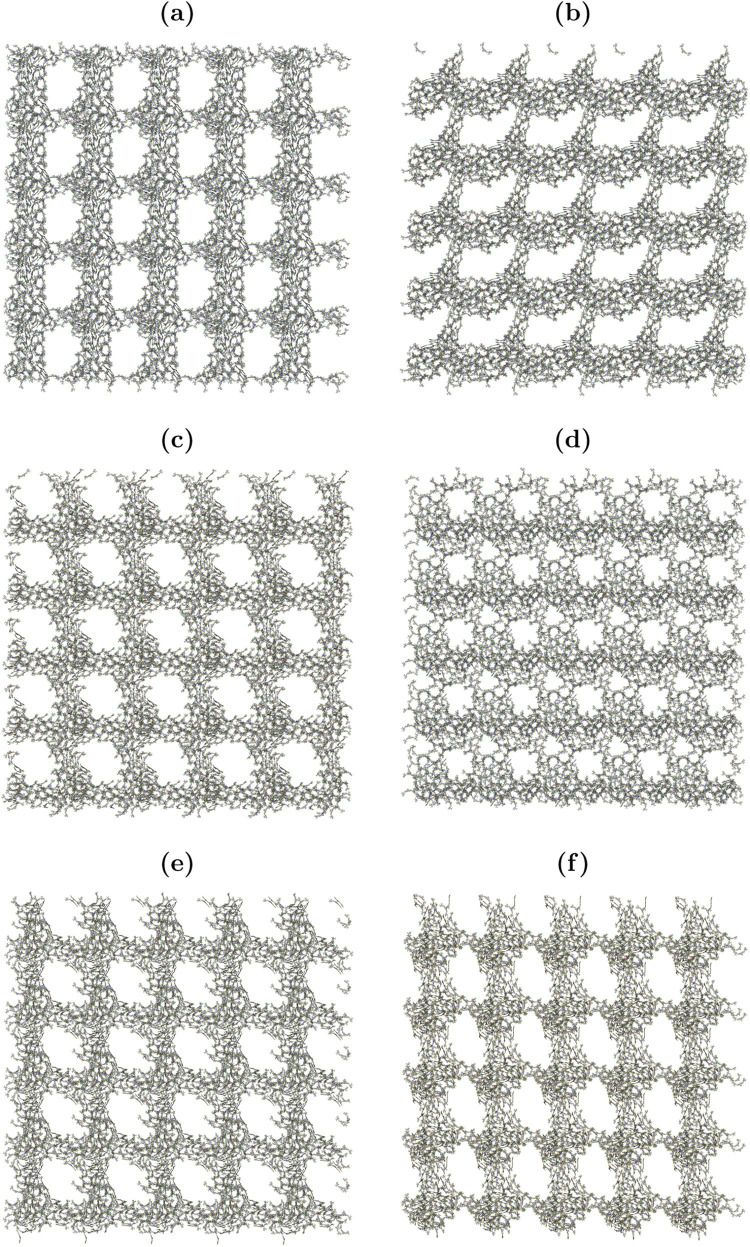
Snapshot of the simulation box with periodicity: (a) Y6–P–IN; (b) Y6–2P-IN; (c) Y6–P–OUT; (d) Y6–2P-OUT; (e) Y6–4P; and (f) Y6. Only the backbones of molecules are displayed.

The MD simulation of solvent evaporation is essentially a nonequilibrium process due to computational cost limitations. It results in the formation of numerous aggregates that start to nucleate at different positions and hardly rearrange to allow for the growth of a more ordered and homogeneous morphology. The consequence is thus the presence of numerous defects in the film structure. We illustrate this behavior in Figure 11, which shows snapshots of the MD simulation box with periodic images, where complete solvent removal resulted in large voids within the simulation box. Ultimately, the presence of those voids might affect the device performance, especially when other species, like donor polymers, are blended with the NFA molecules.^99,100^Figure 11 suggests the presence of a clustered network of acceptor molecules separated by regions of voids on large scales. Eventually, this morphology can be beneficial to improve the device performance since it promotes an improved contact surface for charge separation once the donor material is able to fill those voids. At the same time, it also favors the electron transport throughout the network of clustered acceptor molecules.

**Figure 12:**
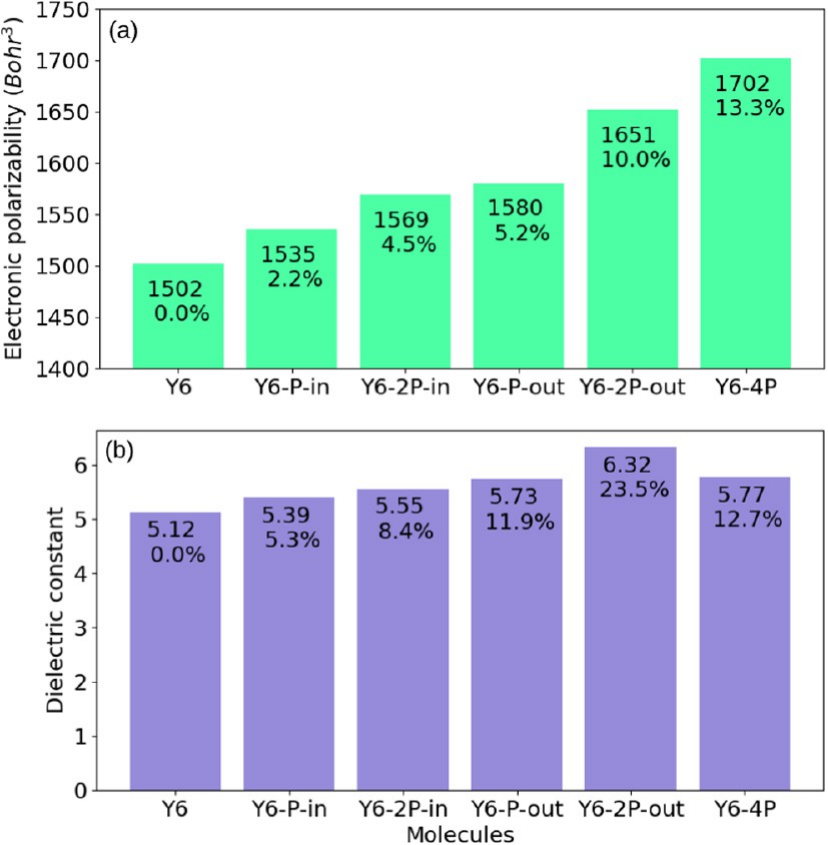
(a) Mean electronic polarizability (α̅). (b) Dielectric constant (ε). Inset: respective values and percentage of variation in relation to Y6.

Finally, Figure 12a shows the average electronic polarizability results (α̅) obtained by DFT from the final configurations of the 100 simulated molecules in the MD. Interestingly, a significant increase in α̅ was observed with the N–to–P modification of the molecules, reaching 10% and 13% for the Y6–2P–out and Y6–4P molecules, respectively. The calculated molecular volume and electronic polarizability were used to obtain the dielectric constant (ε) from eq 2, where the equation states that ε increases with a decrease in volume and an increase in polarizability. Figure 12b shows the dielectric constant determined for each molecule, with all modified molecules experiencing an increase in the dielectric constant, with the highest increase of 23.5% observed for Y6–2P–out. The increase in dielectric constant due to molecular N–to–P substitution is particularly interesting for reducing the exciton binding energy, facilitating the generation of free charges in the material.^14,76^

## 4 Conclusions

In conclusion, our computational study offers detailed insight into the potential of N-to-P substitution in the Y6 molecule, motivated by the critical need to minimize the use of toxic substances, such as malononitrile, in the synthesis of OPV materials. By replacing nitrogen atoms with phosphorus, we sought to address this toxicity issue while investigating the potential to improve the optoelectronic properties of Y6 through this novel substitution strategy. From our key findings presented here, several significant trends could inspire further modifications of Y6 derivatives or other classes of NFAs, paving the way for the development of more sustainable and high-performance organic photovoltaic materials.

In the first part of the study, we analyzed the isolated molecules, focusing on the electronic properties and structural stability of the phosphorus-substituted Y6 derivatives. The results revealed that the introduction of phosphorus considerably altered the molecular electronic distribution, changing the partial charges of the atoms in the modified region. Moreover, the N-to-P substitution provided an increase in electronic delocalization due to the longer P≡C bond compared to N≡C. This leads to a red shift in the absorption spectrum and an increase in oscillator strength, ultimately improving light-harvesting efficiency. It was also observed that the modified molecules exhibit a narrower HOMO–LUMO gap due to a notable stabilization of the HOMO energy level along with a minor destabilization of the LUMO energy. Interestingly, the exciton binding energy was weakened, which is crucial for improving the charge separation. However, a reduction in the torsional energy barrier was observed, suggesting a slight increase in molecular flexibility, especially in molecules with external phosphorus substitution. This raises concerns about potential photostability issues.

In the second part of the study, we examined three dimer configurations (J, C, and H) to assess the influence of phosphorus substitution in the interaction between neighboring molecules. Although the incorporation of phosphorus slightly reduced the quadrupole moment, which impairs intermolecular interactions, the calculated electron transfer rates remained high because of a consistent reduction in the intramolecular reorganization energy.

In the last part of the study, MD simulations provided further insights into the packing and morphological behavior of these materials. The molecular volumes (*V*_M_) calculated from the MD results showed that the Y6–4P molecule exhibited the highest volume, with an increase of approximately 7%. This was expected as a result of the larger van der Waals radius of phosphorus compared to nitrogen. However, for other derivatives with fewer substitutions, the molecular volume remained stable, suggesting compensating factors such as changes in molecular conformations or denser packing.

Regarding conformational variations, no significant changes in the dihedral angle distribution were observed when N atoms were replaced with P. These findings indicate that the minor differences observed in the DFT calculations for isolated molecules are mitigated by intermolecular interactions. The analysis of the radius of gyration (*R*_g_) and end-to-end distance (*d*_ee_) of the alkyl side chains showed no significant structural variations across the different substitutions, indicating that the overall molecular compactness was not strongly influenced by the conformational variations. However, solvent-accessible surface area (SASA) calculations revealed a distinctive trend: Y6 and Y6–4P showed different accessibility, with Y6–4P exhibiting higher SASA due to the exposure of larger phosphorus atoms.

Further analysis of intermolecular interactions shows that van der Waals forces dominate over the Coulomb contribution for the total interaction energy in all systems. Interestingly, the packing efficiency, as evidenced by the number of molecular pairs, was slightly higher for Y6–4P, which promotes better π–π stacking. Snapshots from MD simulations also revealed that Y6–4P forms larger and more tightly packed molecular clusters compared to other systems, also indicating that the minor differences observed in the DFT calculations for dimers are mitigated.

Finally, the morphological analysis of the thin-film structure after simulated solvent evaporation indicated the formation of aggregates driven by intermolecular stacking, leading to a defective network with voids. These voids could potentially enhance device performance by improving charge separation if filled with donor materials in a bulk heterojunction configuration.

Despite these morphological differences, the dielectric constant (ε) analysis showed a notable increase in all phosphorus-substituted systems, with Y6–2P–out and Y6–4P exhibiting the highest increases. This increase in ε is related to a higher electronic polarizability that is important to decrease the exciton binding energy, which favors charge separation and photovoltaic performance.

Overall, a careful optimization of the degree and position of phosphorus substitution could offer a promising pathway for improving the performance of NFAs in OPVs. These results lay the groundwork for further experimental investigations and the design of new materials.

## Data Availability

Data supporting the findings of this study are available in the Supporting Material.

## Supporting Information Available

Bond length alternation; complementary Raman analysis; intramolecular contribution into the Stokes shift; and intramolecular reorganization energy (PDF)

## Dedication

^⊥^ Dedicated to the memory of G.C.
