# Supplementary material for: Impact of Nitrogen-to-Phosphorus Substitution on End Group of the Y6 Nonfullerene Molecule for Organic Photovoltaics: A Comprehensive Modeling Study
Source: ACS Omega. 2025 May 1;10(18):19189–205. doi: 10.1021/acsomega.5c02254 (PMC12079223; doi:10.1021/acsomega.5c02254)
Supplement: Supplementary file 1 — ao5c02254_si_001.pdf [file ao5c02254_si_001.pdf]

Supporting Information: Impact of Nitrogen-to-Phosphorus Substitution on end-group of Y6 Nonfullerene Molecule for Organic Photovoltaics: A Comprehensive Modeling Study

Leandro Benatto,[*,†] Guilherme C. Q. da Silva,[‡] Matheus F. F. das Neves,[¶] João Paulo A. Souza,[§] Luana Wouk,[§] Lucimara Stolz Roman,[¶] Marlus Koehler,[¶] and Graziâni Candiotto[†,‖]

†*Instituto de Física, Universidade Federal do Rio de Janeiro, 21941−909, Rio de Janeiro − RJ, Brazil*

‡*Laboratoire ICB UMR 6303, Université de Bourgogne, 21078 Dijon, France*

¶*Department of Physics, Federal University of Paraná, 81531−980, Curitiba − PR, Brazil*

§*Institute of Physics, University of Brasília, 70919−970, Brasília, DF, Brazil*

‖*Dedicated to the memory of Graziâni Candiotto*

E-mail: lb08@fisica.ufpr.br

Contents

1 Complementary Results **S3**

1.1 Bond Length Alternation S3

1.2 Complementary Raman Analysis S4

1.3 Intramolecular Contribution into the Stokes−Shift S5

1.4 Intramolecular Reorganization Energy S7

References **S7**

1 Complementary Results

1.1 Bond Length Alternation

Table S1: Bond length alternation (BLA) between the bonds C−C and C=C that connect the D and A regions of the molecules.

Molecules	BLA (Å)
Y6	0.052
Y6−P−in	0.054
Y6−2P−in	0.058
Y6−P−out	0.052
Y6−2P−out	0.054
Y6−4P	0.061

1.2 Complementary Raman Analysis

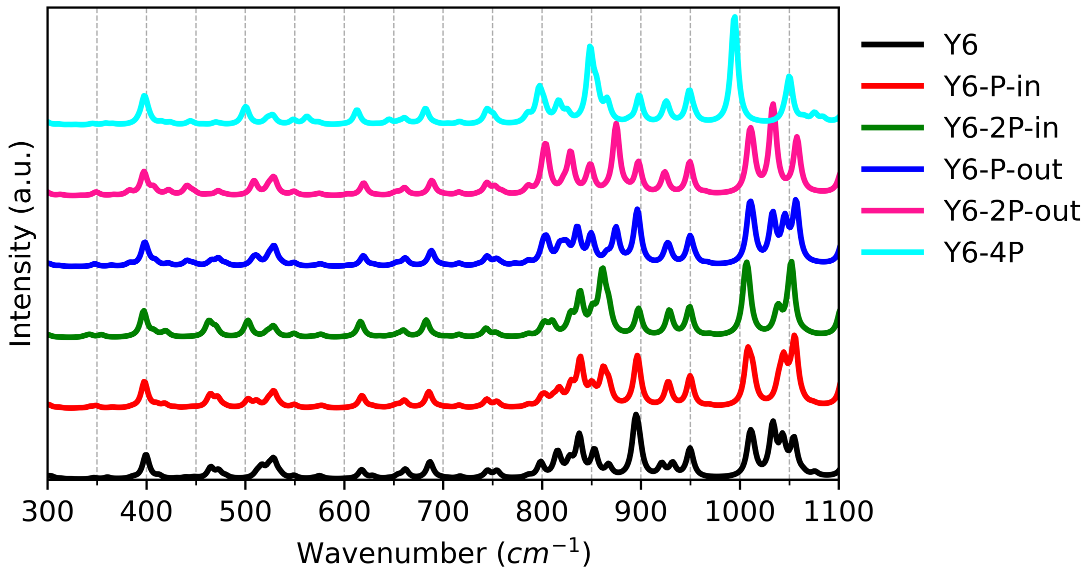

Figure S1: Raman spectra from 300 to 1100 cm^{-1}.

For pure Y6 molecule the $\beta-$CNC band appears in the interval from 380 to 400 cm^{-1} and C−N can be identified in two specific bands around 1070 and 1400 cm^{-1} that corresponds to this stretching vibration and presents lower values as P is introduced in the molecule but don't vanish since there are other functional groups with Carbon and Nitrogen interaction. C=C bond is identified at 1460 cm^{-1} Carbon and Sulfur interaction is observed in C−S stretching vibration that presents a specific Raman band in the region around 620 and 760 cm^{-1}, while for thiophene structure can be associated with the band around 840 cm^{-1} due to ring deformation.[1]

The Raman band associated with $C_\alpha-C_\alpha$ interring stretching can be identified at 1270 cm^{-1} for neat Y6, while it is slightly blue shifted after P substitution. For $C_\beta-C_\beta$ stretching it is identified around 1360 cm^{-1} with small shifts depending on in or out P substitution and this can be associated with different BLA values. Bands associated with Alkene, Phenyl and Carbonyl functional groups are also identified in the respective frequencies: 1550, 1600 and 1705 cm^{-1}.[2]

1.3 Intramolecular Contribution into the Stokes−Shift

Table S2: The vertical absorption energy $E_{S_0 \to S_1}$ calculated on the ground state geometry and the vertical emission energy $E_{S_1 \to S_0}$ calculated on the first excited state geometry (TD-DFT optimization). The energy difference between $E_{S_0 \to S_1}$ and $E_{S_1 \to S_0}$ is considered as an estimation for intramolecular contribution into the Stokes−Shift,[3] $\Delta E^{int}_{rel} = E_{S_0 \to S_1} - E_{S_1 \to S_0}$. All the calculations were done in a vacuum. The experimental result (from Franck Condon analysis) for Y6 in chloroform is 0.103 eV.[3]

Molecules	$E_{S_0 \to S_1}$	$E_{S_1 \to S_0}$	E^{int}_{rel}
Y6	2.22	1.98	0.24
Y6-P-in	2.19	1.95	0.24
Y6-2P-in	2.17	1.92	0.25
Y6-P-out	2.18	1.94	0.24
Y6-2P-out	2.15	1.91	0.24
Y6-4P	2.12	1.85	0.27

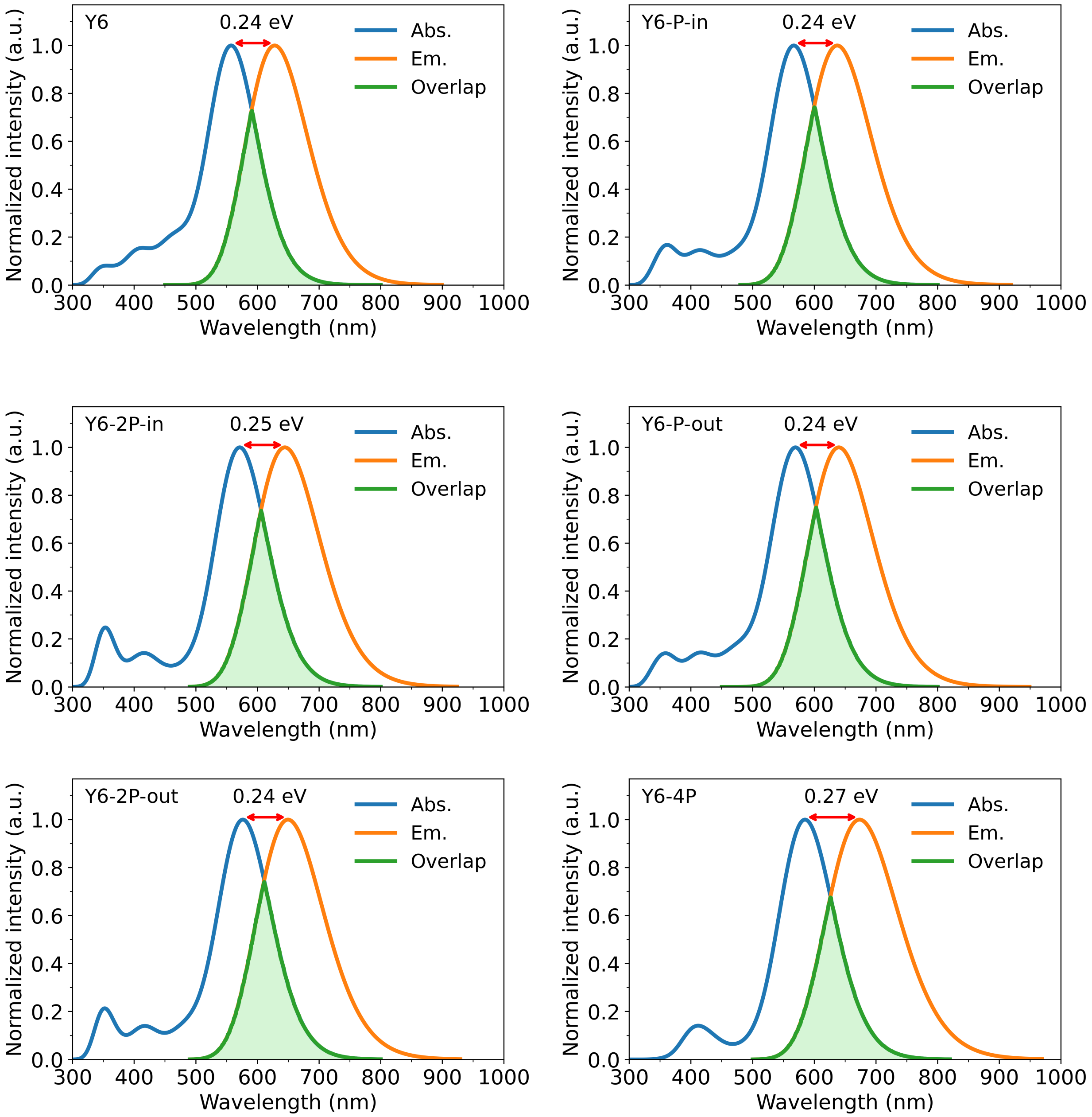

Figure S2: Vertical absorption and emission spectrum of molecules calculated in vacuum. Inset: The double arrow indicates the intramolecular contribution into the Stokes−Shift, $\Delta E_{rel}^{int} = E_{S_0 \rightarrow S_1}$-$E_{S_1 \rightarrow S_0}$.

1.4 Intramolecular Reorganization Energy

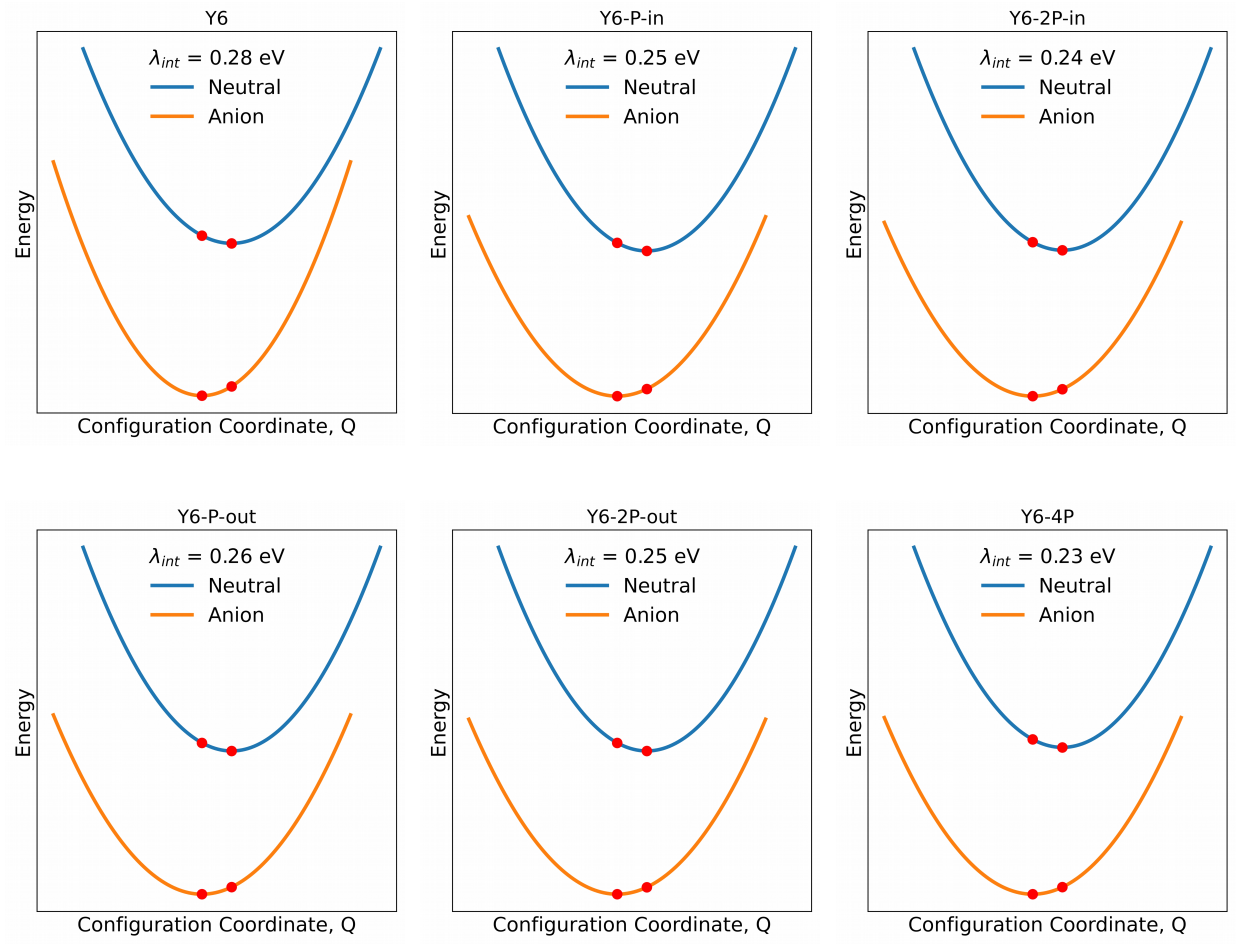

Figure S3: Potential energy surfaces sketch for the neutral and anion structures of the phosphorus-incorporated acceptor molecules. The intramolecular reorganization energy for electron transfer λ_{int} can be calculated by $\lambda_{int} = \left(E_0^- - E_-\right) + \left(E_-^0 - E_0\right)$,[4] where E_0 is the energy of the neutral molecule in neutral geometry, E_- is the energy of the anionic molecule in anionic geometry, E_0^- is the energy of the anionic molecule in neutral geometry, and E_-^0 is the energy of the neutral molecule in anionic geometry.

References

(1) Cordaro, J. G.; Stein, D.; Grützmacher, H. A Synthetic Cycle for the Ruthenium-Promoted Formation of 1 H-Phosphindoles from Phosphaalkynes. *Journal of the American Chemical Society* **2006**, *128*, 14962–14971.

(2) Kurita, E.; Tomonaga, Y.; Matsumoto, S.; Ohno, K.; Matsuura, H. Quantum chemical calculations and vibrational analysis of compounds containing carbon–phosphorus multiple and single bonds. *Journal of Molecular Structure: THEOCHEM* **2003**, *639*, 53–67.

(3) Kashani, S.; Wang, Z.; Risko, C.; Ade, H. Relating reorganization energies, exciton diffusion length and non-radiative recombination to the room temperature UV-vis absorption spectra of NF-SMA. *Mater. Horiz.* **2023**, *10*, 443–453.

(4) Oliveira, E. F.; Lavarda, F. C. Reorganization energy for hole and electron transfer of poly(3-hexylthiophene) derivatives. *Polymer* **2016**, *99*, 105–111.
